# Dietary N-Carbamylglutamate Partially Alleviates High-Starch-Induced Hepatic Oxidative Stress and Glycogenic Hepatopathy in Largemouth Bass (*Micropterus salmoides*)

**DOI:** 10.3390/antiox15060673

**Published:** 2026-05-27

**Authors:** Tao Cheng, Jiandong Chen, Mengfei Liu, Beiping Tan, Shuyan Chi

**Affiliations:** Aquatic Animal Nutrition and Feed Laboratory, Guangdong Ocean University, Zhanjiang 524088, China; chengtao@stu.gdou.edu.cn (T.C.);

**Keywords:** largemouth bass (*Micropterus salmoides*), glycogenic hepatopathy, liver injury, N-carbamylglutamate (NCG), antioxidant defense

## Abstract

High-starch diets are increasingly used in aquafeeds to reduce feed costs, but carnivorous fish such as largemouth bass (*Micropterus salmoides*) have limited capacity to utilize dietary starch and are prone to hepatic metabolic disorders. In the present study, we evaluated whether dietary N-carbamylglutamate (NCG) could alleviate high-starch-induced hepatic oxidative stress and liver injury in largemouth bass. Fish were fed a control diet containing 11.50% starch, a high-starch diet containing 18.00% starch, or a high-starch diet supplemented with 0.15%, 0.20%, or 0.25% NCG for 8 weeks. Compared with the high-starch group, dietary NCG supplementation significantly reduced serum glucose and triglyceride levels, decreased hepatic glycogen and malondialdehyde contents, and increased hepatic superoxide dismutase and glutathione peroxidase activities. NCG also reduced serum alanine aminotransferase and aspartate aminotransferase activities and alleviated hepatic histopathological damage. At the transcriptional level, NCG upregulated genes related to insulin signaling, glycolysis, lipid catabolism, and antioxidant regulation, including *insr*, *irs*, *gk*, *pk*, *atgl*, *hsl*, *ampk*, and *nrf2*, while downregulating the expression of *keap1*, *nf-κB*, *mtor*, and multiple inflammation- and apoptosis-related genes. These changes were accompanied by increased serum nitric oxide levels and improved survival and growth performance under high-starch feeding conditions. Collectively, these results indicate that dietary NCG supplementation attenuates high-starch-induced hepatic oxidative stress and redox-associated liver injury in largemouth bass, which may be associated with the transcriptional modulation of genes related to the AMPK/Nrf2/Keap1 and mTOR/NF-κB signaling pathways.

## 1. Introduction

In recent years, carbohydrate ingredients have become a cost-effective energy source in aquafeeds compared with protein and lipid ingredients. They not only spare protein and lipid resources [1], but also serve as stabilizers and bulking agents in fish feed [2]. Numerous fish studies have indicated that optimal dietary carbohydrate levels are beneficial for feed efficiency and fish growth [3,4,5,6]. However, the ability of fish to utilize starch varies significantly depending on the species of fish [7]. Herbivorous fish are well-suited for diets high in starch [8], whereas carnivorous fish often show impaired health when fed high-starch diets. Excessive dietary starch intake in fish may lead to reduced growth, elevated blood glucose and lipid levels, accumulation of hepatic glycogen and lipids, hepatic damage and immune inhibition [9,10,11,12,13,14].

The largemouth bass (*Micropterus salmoides*), recognized as the fifth major Chinese fish, is highly favored by consumers. Largemouth bass production exceeded 783 thousand tons in 2024 and continues to show an upward trajectory [15]. In current largemouth bass production, the dietary starch level is approximately 10%, and exceeding 15.0% may result in slow growth and compromised liver health in largemouth bass [2,16,17]. In largemouth bass, elevated dietary starch leads to the accumulation of substantial amounts of hepatic glycogen, causing the cytoplasm of hepatocytes to swell markedly. This excess glycogen induces a glassy appearance in the liver, a distinctive characteristic of glycogenic hepatopathy [18], along with inflammation, cirrhosis, fibrosis [19], and a reduction in antioxidant capacity [9].

In previous studies, various additives, such as bile acids [2], olive extract [16], resveratrol inclusion [12], yeast cultures [20], and mannan-oligosaccharides [21], have been incorporated into high-starch diets to alleviate the adverse effects on largemouth bass. However, the exploration of effective and economically feasible additives for improving glucose utilization in this species remains limited.

In the present study, N-carbamylglutamate (NCG) was selected as a novel dietary supplement to mitigate the negative impacts of a high-starch diet on largemouth bass. NCG is a metabolically stable analogue of N-acetylglutamic acid [22] and is known to stimulate endogenous arginine synthesis [23]. It has been widely reported to enhance antioxidant capacity and improve growth performance in various animal species [24,25,26]. Endogenous arginine, a potent stimulator of growth hormone secretion [27], plays a critical role in promoting growth across a broad range of animals [24,28]. In addition, NCG has been shown to facilitate insulin secretion and enhance insulin sensitivity through the activation of endogenous arginine synthesis, thereby regulating glucose metabolism [29,30,31,32]. These physiological functions suggest that NCG may have considerable potential in improving glucose utilization and alleviating the metabolic disorders induced by high dietary starch levels in carnivorous fish.

Therefore, this study aimed to evaluate whether dietary NCG could alleviate high-starch-induced hepatic oxidative stress and glycogenic hepatopathy in largemouth bass, with particular emphasis on antioxidant defense, inflammatory responses, apoptosis, and redox-related signaling pathways.

## 2. Materials and Methods

### 2.1. Experimental Feeds

Five diets were formulated in the present experiment: a control diet containing 11.50% starch (CS), a high-starch diet containing 18.00% starch (HS), and three high-starch diets supplemented with 0.15% (HN0.15), 0.20% (HN0.20), or 0.25% NCG (HN0.25) (Table 1). The formulations of the experimental diets were designed based on previous studies on largemouth bass nutrition and high-starch diet models, with minor modifications [2,9,16,17]. Fish meal and chicken meal were pulverized and passed through a 40-mesh sieve. All ingredients were mixed according to the recipe and then water, soybean oil and fish oil were added to the mixture. Sinking pellets with a diameter of 3 mm were produced using a puffing machine (TSE65S, Beijing Yanggong Machinery Co., Ltd., Beijing, China), which were allowed to air-dry and kept at −20 °C.

### 2.2. Farming Management and Conditions

A total of 450 clinically healthy juvenile largemouth bass of mixed sex were obtained from the Zhanjiang Freshwater Fish Hatchery and acclimated for 14 days with commercial diets. After acclimation, fish with a uniform initial body weight (5.96 ± 0.01 g) were randomly allocated into 15 tanks (300 L; 30 fish per tank) using a random number generator. Each dietary treatment was assigned to three replicate tanks, with 30 fish per tank. The tank, rather than the individual fish, was considered the experimental unit for statistical analysis. The sample size was determined based on previous studies and facility capacity, without a priori power calculation. Fish were maintained in a recirculating aquaculture system with continuous aeration and fed to apparent satiation twice daily under standard culture conditions as described in [33]. During the 8 week growth trial (from June to August 2023), the water quality parameters for largemouth bass included the temperature set at 27.00 ± 2.12 °C, pH value at 7.30 ± 0.25, dissolved oxygen at 6.11 ±1.20 mg/L, ammonia nitrogen at 0.2 ± 0.01 mg/L, and nitrite at 0.03 ± 0.01 mg/L. The photoperiod was 12 L:12 D, with the light period from 07:00 to 19:00. All efforts were made to minimize animal suffering. Fish were monitored daily for health status and abnormal behavior. No unexpected adverse events were observed during the experimental period.

### 2.3. Sample Collection

All largemouth bass were fasted for 24 h prior to sample collection to ensure a consistent physiological status. In each experimental group, fish were first anesthetized with a 0.1 g/L solution of MS-222 (tricaine methanesulfonate). Anesthesia was confirmed by the loss of voluntary movement, absence of response to tactile stimuli (e.g., gentle prodding of the caudal fin), and reduced opercular movement.

After confirming deep anesthesia, euthanasia was performed via exsanguination through massive blood collection from the caudal vein using a syringe. This method involved extracting a sufficient volume of blood to induce a rapid and irreversible loss of vital signs, ensuring minimal suffering and compliance with humane endpoints for aquatic animals. The collected blood samples were deposited into 1.5 mL centrifuge tubes and stored at 4 °C for 24 h to allow clotting.

Following euthanasia, all fish were counted and weighed. Three fish per tank were randomly selected for body composition analysis. Another three fish per tank were measured for body weight, visceral weight, and liver weight to calculate the viscerosomatic index (VSI) and hepatosomatic index (HSI). Serum was separated by centrifugation at 3200× *g* for 8 min at 4 °C and stocked at −80 °C for the determination of serum biochemical indices.

Livers from seven fish per tank were rapidly dissected, flash-frozen in liquid nitrogen to preserve enzymatic activity and RNA integrity, and transferred to −80 °C for the analysis of hepatic biochemical indices and mRNA expression. Livers from two fish per tank were immediately immersed in 4% paraformaldehyde solution for histological analysis (Oil Red O staining and hematoxylin–eosin staining). After sample collection, the remaining largemouth bass carcasses and biological waste were securely packed in biohazard bags and transferred for centralized incineration in accordance with institutional biosafety and animal care regulations.

All experimental procedures involving fish were approved by the Experimental Animal Ethics Committee of Guangdong Ocean University (approval number: GDOUIACUC-2022-A0108; approval date: 5 June 2022) and strictly adhered to the ethical standards for the use and care of laboratory animals.

### 2.4. Proximate Composition Analysis of Feeds and Fish, and Liver Section Preparation

The proximate compositions of the experimental feeds and fish bodies were analyzed following the standardized methods detailed in Table 2.

All stained sections were photographed under identical microscope, magnification, illumination, and camera settings. Histological images were analyzed using ImageJ 1.8.0 software (NIH, Bethesda, MD, USA). Image capture and quantitative analysis were performed before the treatment identities of the sections were checked. After image quantification was completed, the corresponding treatment groups were identified for statistical analysis. To minimize subjective bias, all images were quantified using the same predefined analysis procedure and fixed thresholding criteria.

For each tank, liver samples from two fish were used for histological analysis. For each fish, one representative section was analyzed, and five randomly selected non-overlapping fields were captured from each section.

For HE-stained sections, vacuolated regions were identified as unstained or weakly stained intracellular empty areas within hepatocytes. The same thresholding criteria were applied to all HE-stained images. The relative vacuolated area was calculated as vacuolated area/total tissue area × 100.

For Oil Red O-stained sections, Oil Red O-positive areas were identified according to red staining using a fixed color threshold, which was applied consistently to all images. Lipid staining intensity was quantified as the integrated optical density (IOD) of Oil Red O-positive staining normalized to the analyzed tissue area and expressed as IOD/area.

For each fish, the mean value of all analyzed fields was used as the representative value, and values from fish within the same tank were averaged to obtain one tank mean before statistical analysis.

### 2.5. Hepatic and Serum Biochemical Indices

Serum samples were thawed at 4 °C before biochemical analysis. Serum total triglyceride (TG), glucose (GLU), nitric oxide (NO) levels, and alanine aminotransferase (ALT) and aspartate aminotransferase (AST) activities were measured using commercial assay kits purchased from Nanjing Jiancheng Bioengineering Institute Nanjing, China according to the manufacturer’s instructions. The catalog numbers of the kits were as follows: TG A110-1-1, GLU A154-1-1, NO A013-2-1, ALT C009-2-1, and AST C010-2-1. Serum TG and GLU were determined using enzymatic colorimetric methods. Serum NO was determined using a nitrate reductase-based colorimetric method. Serum ALT and AST activities were measured based on the enzymatic formation of pyruvate or oxaloacetate from their corresponding substrates, followed by colorimetric detection. Absorbance was measured at the wavelengths specified in the corresponding kit protocols.

For hepatic biochemical analysis, liver samples were rinsed with ice-cold physiological saline to remove blood residues, weighed, and homogenized in ice-cold saline. The homogenates were centrifuged at 3370× *g* for 10 min at 4 °C, and the supernatants were collected for biochemical assays. Hepatic glycogen, malondialdehyde MDA, superoxide dismutase SOD, and glutathione peroxidase GSH-PX were measured using commercial kits from the Nanjing Jiancheng Bioengineering Institute Nanjing, China according to the manufacturer’s protocols. The catalog numbers were as follows: glycogen A043-1-1, MDA A003-1-2, SOD A001-1-1, and GSH-PX A005-2-1. Hepatic glycogen was determined using a colorimetric assay after hydrolysis. MDA content was measured using the thiobarbituric acid reactive substances (TBARSs) method. SOD activity was determined based on the inhibition of superoxide anion-mediated chromogenic reactions, whereas GSH-PX activity was measured based on the enzymatic reduction of peroxide coupled with glutathione consumption. Absorbance was recorded at the wavelengths specified in the corresponding kit protocols. Results were expressed according to the units defined by each assay kit.

The protein concentration in the liver homogenate supernatants was determined using a BCA protein assay kit A045-4-2, Nanjing Jiancheng Bioengineering Institute, Nanjing, China according to the manufacturer’s instructions. The hepatic MDA content, SOD activity, and GSH-PX activity were normalized to the corresponding protein concentrations and expressed as nmol/mg protein, U/mg protein, and U/mg protein, respectively, whereas the hepatic glycogen content was calculated based on the initial liver wet weight and expressed as mg/g wet tissue.

### 2.6. Real-Time Fluorescence Quantitative PCR (qRT-PCR) Analysis

qRT-PCR was used to quantify the transcript levels of genes associated with arginine synthesis, glucose metabolism, lipid metabolism, antioxidant response, inflammation, apoptosis, and signaling-related responses. The total RNA was extracted from liver or intestinal samples using TRIzol reagent (Invitrogen, Carlsbad, CA, USA) according to the manufacturer’s instructions. The RNA concentration and purity were determined using a spectrophotometer (ND-1000, Thermo Fisher Scientific, Waltham, MA, USA). Samples with A260/A280 ratios between 1.8 and 2.1 and A260/A230 ratios above 2.0 were used for subsequent analysis. RNA integrity was verified by electrophoresis on a 1.0% agarose gel, and only RNA samples showing clear 28S and 18S rRNA bands without obvious degradation were used for cDNA synthesis.

Complementary DNA (cDNA) was synthesized using the PrimeScript RT reagent kit (Takara, Kusatsu, Shiga, Japan). qRT-PCR amplification was performed using the SYBR Premix Ex Taq kit (Takara, Kusatsu, Shiga, Japan) with the gene-specific primers listed in Table 3. The reaction protocol was as follows: initial denaturation at 95 °C for 30 s, followed by 40 cycles of denaturation at 95 °C for 5 s, annealing at 55 °C for 30 s, and extension at 72 °C for 60 s. A melting curve analysis was performed after amplification to verify the specificity of each PCR product.

Primer specificity was confirmed by melting curve analysis, and only primer pairs showing a single melting peak without obvious non-specific amplification were used for subsequent analysis. The primer amplification efficiency was evaluated using standard curves generated from serially diluted cDNA samples. The amplification efficiencies of all primer pairs ranged from 90% to 110%. The primer sequences, accession numbers, amplicon sizes, and amplification efficiencies are shown in Table 3.

β-actin and ef1α were used as internal reference genes. The average Ct value of β-actin and ef1α was used as the reference Ct value for the ΔCt calculation, and the relative mRNA expression levels of target genes were calculated using the 2^−ΔΔCt^ method [38].

### 2.7. Data Statistics and Analysis

All statistical analyses were performed using SPSS 19.0 software (SPSS Inc., Chicago, IL, USA). Data are presented as means ± standard error of the mean (SEM). The tank, rather than the individual fish, was considered the experimental unit for all statistical analyses, with three replicate tanks per dietary treatment, i.e., n = 3.

The normality and homogeneity of variance were assessed using the Shapiro–Wilk test and Levene’s test, respectively. Differences among the five dietary treatments were analyzed by one-way analysis of variance (ANOVA). When significant differences were detected, Tukey’s post hoc multiple comparison test was used to compare treatment means. Statistical significance was set at *p* < 0.05.

Pearson correlation analysis was used to evaluate associations among hepatic biochemical indices, antioxidant enzyme activities, and gene expression levels. To avoid pseudoreplication, the correlation analysis was conducted using tank-level mean values from all 15 tanks, rather than individual fish values or treatment means. These correlation results were interpreted as associations rather than evidence of causal relationships. The correlation heatmap was generated using an online visualization platform (https://www.bioinformatics.com.cn).

## 3. Results

### 3.1. Growth Performance

**Figure 1:**
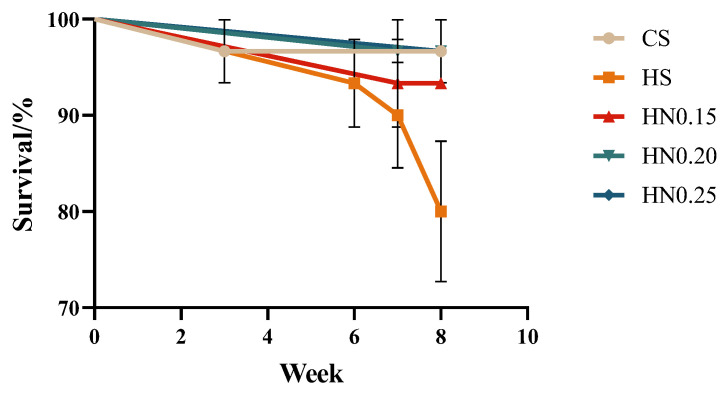
Effects of NCG added in a high-starch diet on the survival rate curve of largemouth bass.

As shown in Table 4, compared with the CS group, the FBW and SGR were significantly reduced in the HS, HN0.15, HN0.20, and HN0.25 groups (*p* < 0.05). However, the FBW and SGR were significantly higher in the HN0.15 and HN0.20 groups than in the HS and HN0.25 groups (*p* < 0.05). The FBW and SGR in the HN0.25 group were also significantly higher than those in the HS group, but remained lower than those in the HN0.15 and HN0.20 groups (*p* < 0.05). Compared with the CS group, the WGR was significantly reduced in the HS and NCG-supplemented groups, whereas the WGR was significantly higher in the HN0.15, HN0.20, and HN0.25 groups than in the HS group (*p* < 0.05). The FI was significantly lower in the HN0.15 and HN0.20 groups than in the HS group (*p* < 0.05), while no significant differences in the FI were observed among the CS, HS, and HN0.25 groups according to the lettering assignments. The survival rates in the HN0.15, HN0.20, and HN0.25 groups were not significantly different from that in the CS group (*p* > 0.05), but were significantly higher than that in the HS group (*p* < 0.05). As shown in Figure 1, mortality in the HS group mainly occurred during the last three weeks of the feeding trial.

### 3.2. Fish Composition

As shown in Table 5, compared with the CS group, the whole-body crude protein content was significantly increased in the HN0.15, HN0.20, and HN0.25 groups (*p* < 0.05). Compared with the HS group, the crude protein content was significantly higher in the HN0.15 and HN0.20 groups (*p* < 0.05), whereas no significant difference was observed between the HS and HN0.25 groups (*p* > 0.05).

### 3.3. Viscerosomatic Index and Hepatosomatic Index

**Figure 2:**
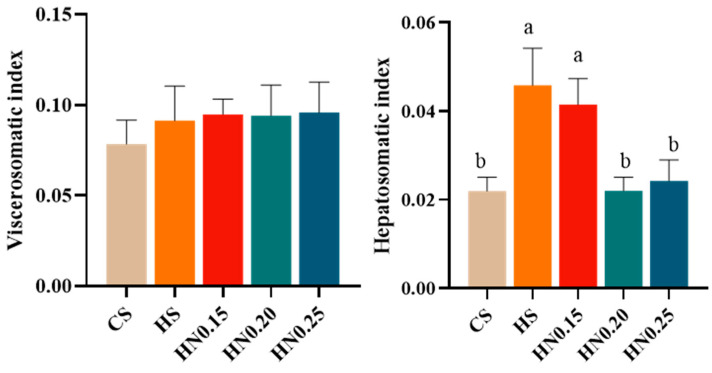
Effects of NCG added in a high-starch diet on the viscerosomatic index and hepatosomatic index in largemouth bass. Vertical bars represent the mean ± SEM (n = 3). Data with different letters were significantly different between all treatment groups (*p* < 0.05). One-way ANOVA results: Viscerosomatic index, F(4, 10) = 1.93; Hepatosomatic index, F(4, 10) = 2.28.

As shown in Figure 2, compared with the CS group, the hepatosomatic index was significantly increased in the HS and HN0.15 groups (*p* < 0.05). No significant difference in the hepatosomatic index was observed between the HS and HN0.15 groups (*p* > 0.05). In addition, the hepatosomatic index in the HN0.20 and HN0.25 groups did not differ significantly from that in the CS group (*p* > 0.05).

### 3.4. Serum Biochemical Indexes

**Figure 3:**
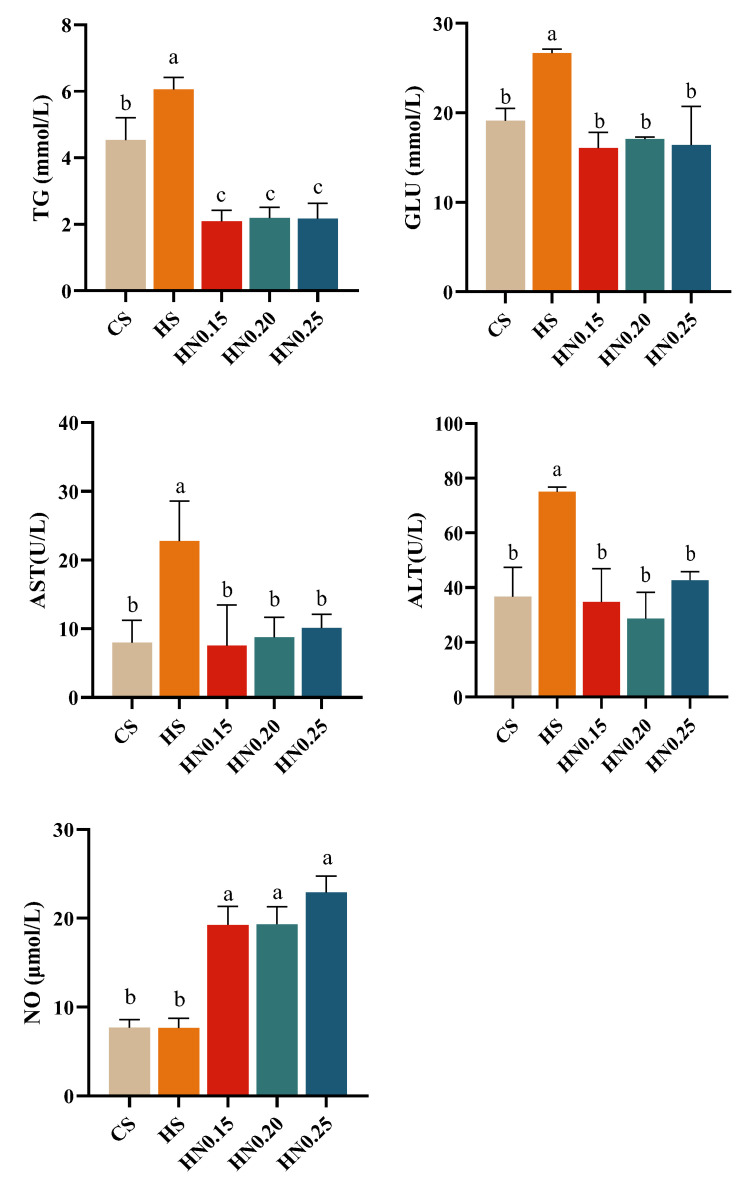
Effects of NCG added in a high-starch diet on the serum total triglyceride (TG), glucose (GLU), aspartate aminotransferase (AST), alanine aminotransferase (ALT), and nitric oxide (NO) in largemouth bass. Vertical bars represent the mean ± SEM (n = 3). Data with different letters were significantly different between all treatment groups (*p* < 0.05). One-way ANOVA results: TG, F(4, 10) = 49.37; GLU, F(4, 10) = 12.11; AST, F(4, 10) = 6.71; ALT, F(4, 10) = 13.63; NO, F(4, 10) = 57.34.

As shown in Figure 3, compared with the CS group, serum TG levels were significantly increased in the HS group but significantly decreased in the HN0.15, HN0.20, and HN0.25 groups (*p* < 0.05). Compared with the HS group, serum TG levels were significantly reduced in all NCG-supplemented groups (*p* < 0.05). Serum GLU levels and AST and ALT activities were significantly increased in the HS group compared with the CS group (*p* < 0.05), whereas no significant differences were observed between the CS group and the NCG-supplemented groups (*p* > 0.05). Compared with the HS group, serum GLU levels and AST and ALT activities were significantly lower in the HN0.15, HN0.20, and HN0.25 groups (*p* < 0.05). Compared with both the CS and HS groups, serum NO concentrations were significantly increased in the HN0.15, HN0.20, and HN0.25 groups (*p* < 0.05).

### 3.5. Arginine Synthesis Genes in the Intestine

**Figure 4:**
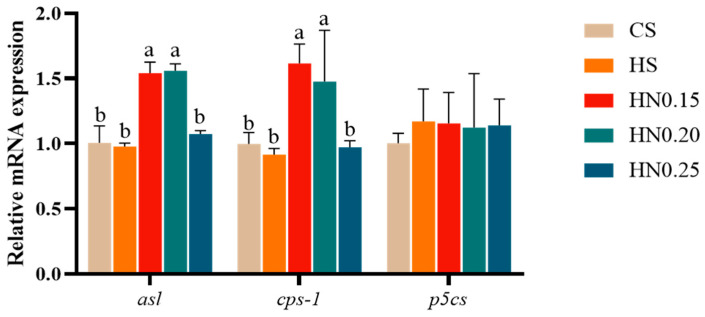
Effects of NCG added in a high-starch diet on the relative expression of arginine synthesis mRNAs in the intestine of largemouth bass. *asl*, arginine succinic acid lyase; *cps-1*, carbamyl phosphate synthase I; *p5cs*, pyrrolin-5-carboxylic acid synthetase. Vertical bars represent the mean ± SEM (n = 3). Data with different letters were significantly different between all treatment groups (*p* < 0.05). One-way ANOVA results: *asl*, F(4, 10) = 45.83; *cps-1*, F(4, 10) = 8.374; *p5cs*, F(4, 10) = 0.199.

As shown in Figure 4, compared with the CS group, the intestinal mRNA expression levels of *asl* and *cps-1* were significantly increased in the HN0.15 and HN0.20 groups (*p* < 0.05), whereas no significant differences were observed in the HS and HN0.25 groups (*p* > 0.05). Compared with the HS group, the *asl* and *cps-1* expression levels were significantly higher in the HN0.15 and HN0.20 groups (*p* < 0.05), but not in the HN0.25 group (*p* > 0.05). The intestinal mRNA expression level of *p5cs* did not differ significantly among the five groups (*p* > 0.05).

### 3.6. Hepatic Glycogen and TG

**Figure 5:**
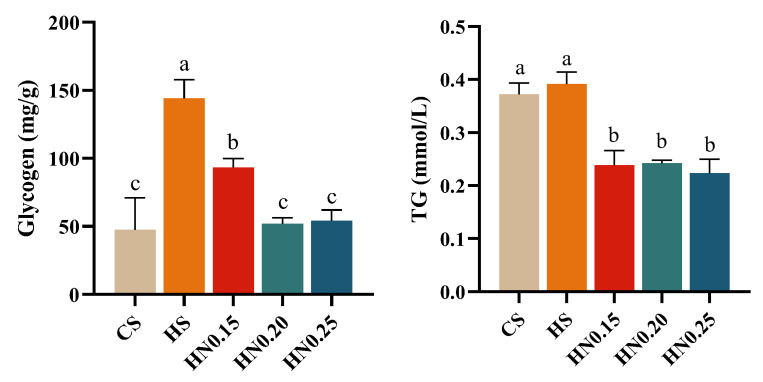
Effects of NCG added in a high-starch diet on the liver glycogen and total triglyceride (TG) in largemouth bass. Vertical bars represent the mean ± SEM (n = 3). Data with different letters were significantly different between all treatment groups (*p* < 0.05). One-way ANOVA results: Glycogen, F(4, 10) = 29.31; TG, F(4, 10) = 40.72.

As shown in Figure 5, compared with the CS group, the hepatic glycogen content did not differ significantly in the HN0.20 and HN0.25 groups (*p* > 0.05). However, the hepatic glycogen content was significantly lower in the HN0.20 and HN0.25 groups than in the HS and HN0.15 groups (*p* < 0.05). Compared with the CS group, hepatic TG levels were not significantly changed in the HS group (*p* > 0.05), but were significantly reduced in the HN0.15, HN0.20, and HN0.25 groups (*p* < 0.05). Similarly, hepatic TG levels were significantly lower in all NCG-supplemented groups than in the HS group (*p* < 0.05).

### 3.7. Hepatic Antioxidant Enzyme Activity

**Figure 6:**
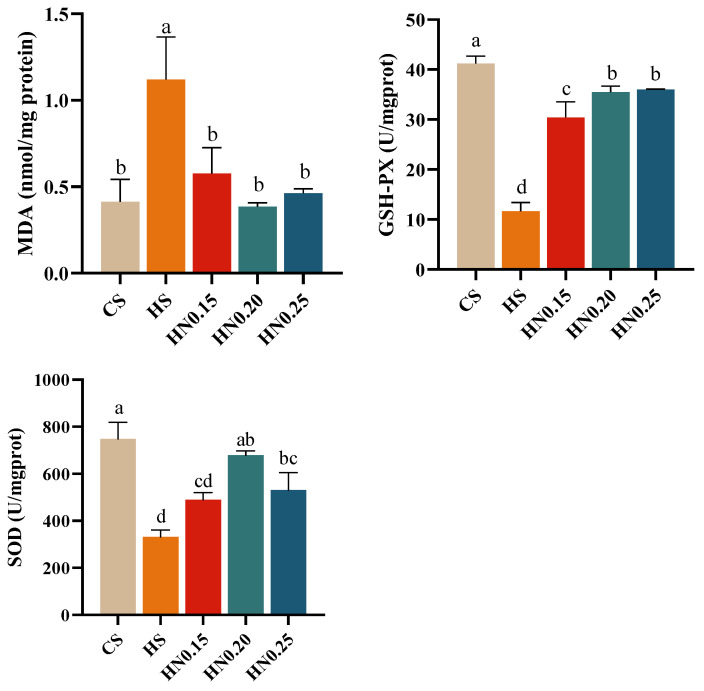
Effects of NCG added in a high-starch diet on the liver malondialdehyde (MDA), superoxide dismutase (SOD) and glutathione peroxidase (GSH-PX) in largemouth bass. Vertical bars represent the mean ± SEM (n = 3). Data with different letters were significantly different between all treatment groups (*p* < 0.05). One-way ANOVA results: MDA, F(4, 10) = 13.91; GSH-PX, F(4, 10) = 119.5; SOD, F(4, 10) = 32.05.

As shown in Figure 6, compared with the CS group, the hepatic MDA content was significantly increased in the HS group (*p* < 0.05). Dietary NCG supplementation significantly reduced the hepatic MDA content in the HN0.15, HN0.20, and HN0.25 groups compared with the HS group (*p* < 0.05), with no significant differences observed between the NCG-supplemented groups and the CS group (*p* > 0.05).

Compared with the CS group, the hepatic SOD and GSH-PX activities were significantly decreased in the HS group (*p* < 0.05). Dietary NCG supplementation significantly increased the hepatic SOD and GSH-PX activities in the HN0.15, HN0.20, and HN0.25 groups compared with the HS group (*p* < 0.05), although these activities remained significantly lower than those in the CS group (*p* < 0.05).

### 3.8. Expression of Hepatic Apoptosis Genes

**Figure 7:**
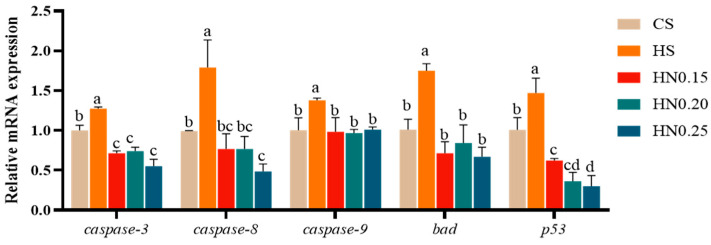
Effects of NCG added in a high-starch diet on the relative expression of apoptosis-related mRNAs in the liver of largemouth bass. *bad*, Bcl-2-associated death protein; *p53*, Recombinant tumor protein P53. Vertical bars represent the mean ± SEM (n = 3). Data with different letters were significantly different between all treatment groups (*p* < 0.05). One-way ANOVA results: *caspase-3*, F(4, 10) = 77.43; *caspase-8*, F(4, 10) = 119.5; *caspase-9*, F(4, 10) = 5.80; *bad*, F(4, 10) = 26.28; *p53*, F(4, 10) = 39.35.

As shown in Figure 7, compared with the CS group, the hepatic mRNA expression levels of *caspase-3*, *p53*, *caspase-8*, *bad*, and *caspase-9* were significantly increased in the HS group (*p* < 0.05). Dietary NCG supplementation significantly reduced the expression levels of these apoptosis-related genes in the HN0.15, HN0.20, and HN0.25 groups compared with the HS group (*p* < 0.05).

### 3.9. Expression of Hepatic Inflammatory Genes

**Figure 8:**
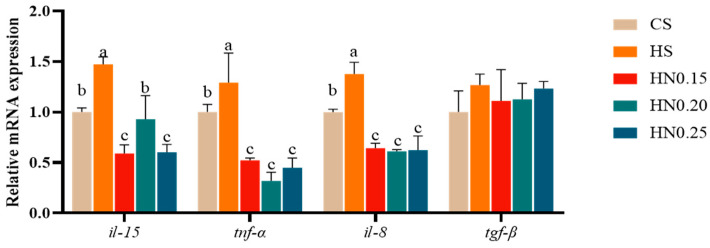
Effects of NCG added in a high-starch diet on the relative expression of inflammation-related mRNAs in the liver of largemouth bass. *il-15*, interleukin-15; *tnf-α*, tumor necrosis factor alpha; *tgf-β*,transforming growth factor-β; *il-8*, interleukin-8. Vertical bars represent the mean ± SEM (n = 3). Data with different letters were significantly different between all treatment groups (*p* < 0.05). One-way ANOVA results: *il-15*, F(4, 10) = 26.01; *tnf-α*, F(4, 10) = 23.65; *il-8*, F(4, 10) = 45.75; *tgf-β*, F(4, 10) = 0.91.

As shown in Figure 8, compared with the CS group, the hepatic mRNA expression levels of *tnf-α* and *il-8* were significantly increased in the HS group but significantly decreased in the HN0.15, HN0.20, and HN0.25 groups (*p* < 0.05). Compared with the HS group, dietary NCG supplementation significantly reduced the hepatic *tnf-α* and *il-8* expression levels in the HN0.15, HN0.20, and HN0.25 groups (*p* < 0.05). Hepatic *il-15* expression was significantly increased in the HS group compared with the CS group, whereas dietary NCG supplementation significantly decreased *il-15* expression compared with the HS group (*p* < 0.05). No significant difference in hepatic *tgf-β* expression was observed among the five groups (*p* > 0.05).

### 3.10. Expression of Genes Related to Hepatic Lipid Metabolism

**Figure 9:**
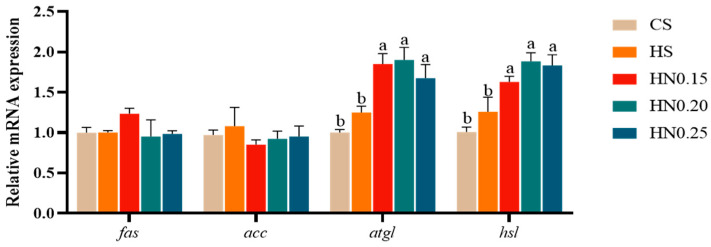
Effects of NCG added in a high-starch diet on the relative expression of lipid metabolism-related mRNAs in the liver of largemouth bass. *fas*, fatty acid synthase; *acc*, acetyl-CoA carboxylase; *atgl*, adipose triglyceride lipase; *hsl*, hormone-sensitive lipase. Vertical bars represent the mean ± SEM (n = 3). Data with different letters were significantly different between all treatment groups (*p* < 0.05). One-way ANOVA results: *fas*, F(4, 10) = 3.58; *acc*, F(4, 10) = 1.19; *atgl*, F(4, 10) = 29.30; *hsl*, F(4, 10) = 30.67.

As shown in Figure 9, compared with the CS group, the hepatic mRNA expression levels of *atgl* and *hsl* were not significantly changed in the HS group (*p* > 0.05), but were significantly increased in the HN0.15, HN0.20, and HN0.25 groups (*p* < 0.05). Compared with the HS group, dietary NCG supplementation significantly upregulated hepatic *atgl* and *hsl* expression levels in the HN0.15, HN0.20, and HN0.25 groups (*p* < 0.05).

### 3.11. Expression of Genes Related to Hepatic Glycometabolism

**Figure 10:**
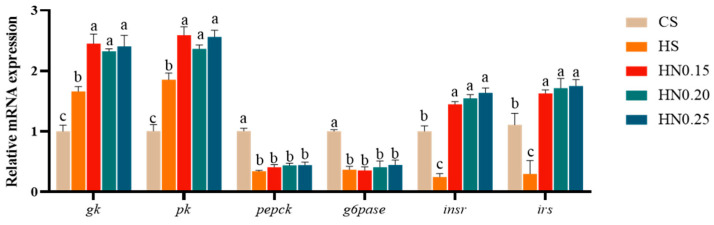
Effects of NCG added in a high-starch diet on the relative expression of glucose metabolism mRNAs in the liver of largemouth bass. *gk*, glucokinase; insr, insulin receptor; *irs*, insulin receptorsubstrate; *pk*, pyruvate kinase; *pepck*, phosphoenolpyruvate carboxykinase; *g6pase*, glucose-6-phosphatase. Vertical bars represent the mean ± SEM (n = 3). Data with different letters were significantly different between all treatment groups (*p* < 0.05). One-way ANOVA results: *gk*, F(4, 10) = 77.74; *pk*, F(4, 10) = 116.79; *pepck*, F(4, 10) = 149.31; *g6pase*, F(4, 10) = 44.55; *insr*, F(4, 10) = 215.54; *irs*, F(4, 10) = 45.48.

As shown in Figure 10, compared with the CS group, the hepatic mRNA expression levels of *gk* and *pk* were significantly increased in the HS group and were further increased in the HN0.15, HN0.20, and HN0.25 groups (*p* < 0.05). Compared with the HS group, dietary NCG supplementation significantly increased hepatic *gk* and *pk* expression levels in all NCG-supplemented groups (*p* < 0.05). In contrast, compared with the CS group, hepatic *insr* and *irs* expression levels were significantly decreased in the HS group but significantly increased in the HN0.15, HN0.20, and HN0.25 groups (*p* < 0.05). Dietary NCG supplementation also significantly increased hepatic *insr* and *irs* expression levels compared with the HS group (*p* < 0.05). Furthermore, compared with the CS group, hepatic *pepck* and *g6pase* expression levels were significantly decreased in the HS and all NCG-supplemented groups (*p* < 0.05).

### 3.12. Expression of Genes Related to the AMPK Pathway

**Figure 11:**
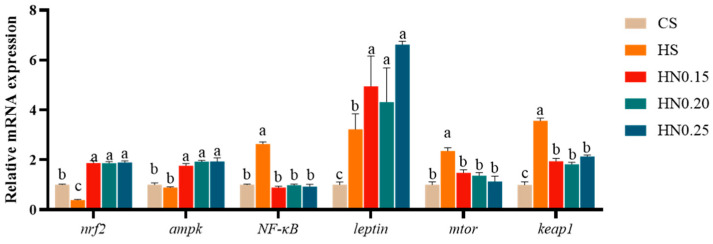
Effects of NCG added in a high-starch diet on the relative expression of AMPK-related pathways’ mRNAs in the liver of largemouth bass. *nrf2*, nuclear factor erythroid-2; *ampk*, Adenosine 5‘-monophosphate-activated protein kinase; *NF-κB*, nuclear factor kappa-B; *keap1*, kelch-like ECH-associated protein-1; *mtor*, mammalian target of rapamycin. Vertical bars represent the mean ± SEM (n = 3). Data with different letters were significantly different between all treatment groups (*p* < 0.05). One-way ANOVA results: *nrf2*, F(4, 10) = 381.13; *ampk*, F(4, 10) = 108.41; *NF-κB*, F(4, 10) = 447.37; *leptin*, F(4, 10) = 17.43; *mtor*, F(4, 10) = 41.94; *keap1*, F(4, 10) = 259.42.

As shown in Figure 11, compared with the CS group, hepatic *nrf2* expression was significantly decreased in the HS group but significantly increased in the HN0.15, HN0.20, and HN0.25 groups (*p* < 0.05). Compared with the HS group, dietary NCG supplementation significantly upregulated hepatic *nrf2* expression in all NCG-supplemented groups (*p* < 0.05). Hepatic *ampk* expression did not differ significantly between the CS and HS groups (*p* > 0.05), but was significantly increased in the HN0.15, HN0.20, and HN0.25 groups compared with both the CS and HS groups (*p* < 0.05). Compared with the CS group, hepatic *NF-κB* and mtor expression levels were significantly increased in the HS group (*p* < 0.05), whereas no significant differences were observed between the CS group and the NCG-supplemented groups (*p* > 0.05). Dietary NCG supplementation significantly reduced hepatic *NF-κB* and *mtor* expression levels compared with the HS group (*p* < 0.05). Compared with the CS group, hepatic *leptin* expression was significantly increased in the HS group and was further increased in the HN0.15, HN0.20, and HN0.25 groups (*p* < 0.05). Compared with the HS group, hepatic *leptin* expression was significantly higher in all NCG-supplemented groups (*p* < 0.05). Hepatic *keap1* expression was significantly increased in the HS group compared with the CS group (*p* < 0.05), whereas dietary NCG supplementation significantly reduced *keap1* expression compared with the HS group (*p* < 0.05), although the levels in the NCG-supplemented groups remained significantly higher than those in the CS group (*p* < 0.05).

### 3.13. Integrated Analysis of Physiological and Biochemical Indicators with Gene Expression in the Liver

**Figure 12:**
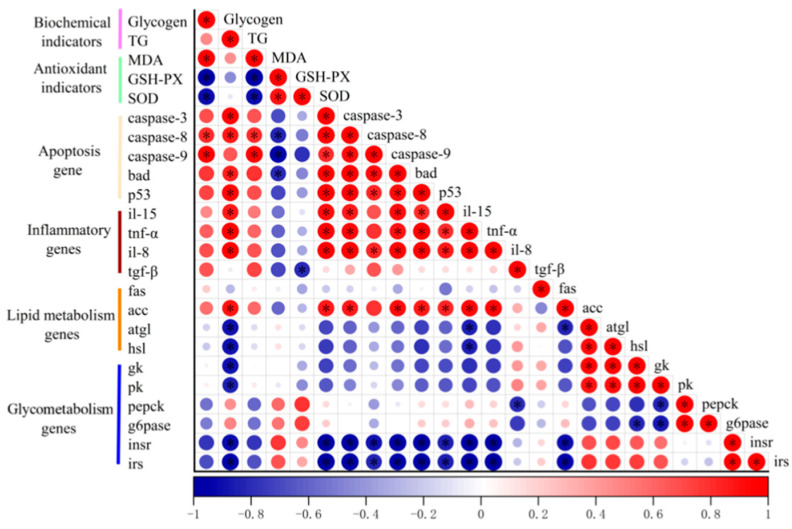
Correlation analysis of liver lipid deposition, antioxidant capacity, and the transcription of genes participating in glucose, apoptosis, inflammatory and lipid metabolism. Color depth signifies the magnitude of the correlation, with blue indicating a positive correlation and red indicating a negative correlation. The size of the circle is proportional to the value. An asterisk (∗) denotes a significance level of *p* < 0.05. Pearson correlation analysis was performed using tank-level mean values from all 15 tanks.

As shown in Figure 12, hepatic glycogen and TG contents were positively correlated with MDA content and with the expression of apoptosis-related genes, including *caspase-3*, *caspase-8*, *caspase-9*, *bad*, and *p53*, as well as pro-inflammatory genes, including *il-15*, *tnf-α*, and *il-8* (*p* < 0.05). In contrast, hepatic SOD and GSH-PX activities were negatively correlated with hepatic glycogen, TG, MDA, and the expression of apoptosis- and inflammation-related genes (*p* < 0.05).

Hepatic SOD and GSH-PX activities were positively correlated with the expression of genes associated with glycolysis, insulin signaling, and lipid catabolism, including *gk*, *pk*, *insr*, *irs*, *atgl*, *hsl*, *ampk*, and *leptin* (*p* < 0.05). These correlation results indicate associations among hepatic glycogen accumulation, oxidative stress, inflammatory and apoptotic responses, and glycolipid metabolism-related gene expression.

### 3.14. Hepatic Morphology

**Figure 13:**
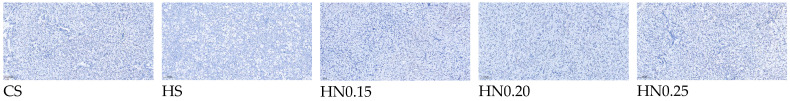
Largemouth bass liver Oil Red O-stained sections (×100).

**Figure 14:**
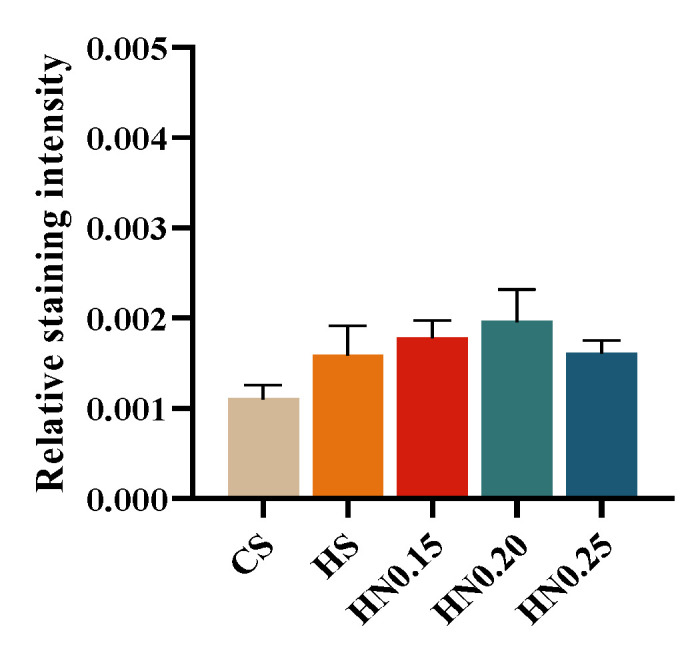
Effect of NCG added in a high-starch diet on lipid accumulation in the liver tissue of largemouth bass. Values are presented as means ± SEM of three replicates. Data with different letters were significantly different between all treatment groups (*p* < 0.05). One-way ANOVA results: F(4, 10) = 3.44.

Oil Red O-positive lipid droplets were stained red. As shown in Figure 13, no obvious lipid droplet deposition was observed in liver sections from any treatment group. Quantitative analysis of Oil Red O staining intensity showed no significant difference among the five groups (*p* > 0.05; Figure 14).

**Figure 15:**
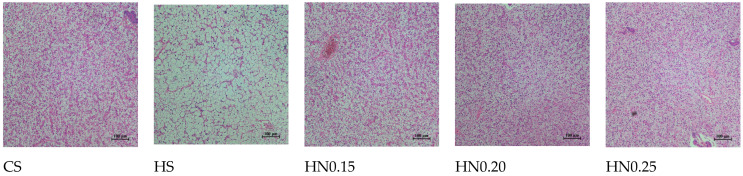
Largemouth bass liver hematoxylin–eosin stained sections (×100).

**Figure 16:**
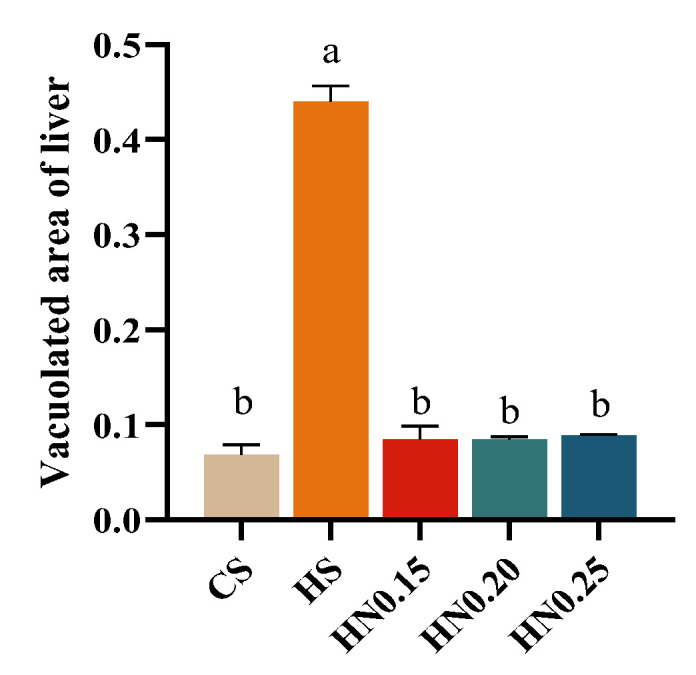
Effect of NCG added in a high-starch diet on the vacuolation of liver cells in largemouth bass. Values are presented as means ± SEM of three replicates. Data with different letters were significantly different between all treatment groups (*p* < 0.05). One-way ANOVA results: F(4, 10) = 439.34.

**Figure 17:**
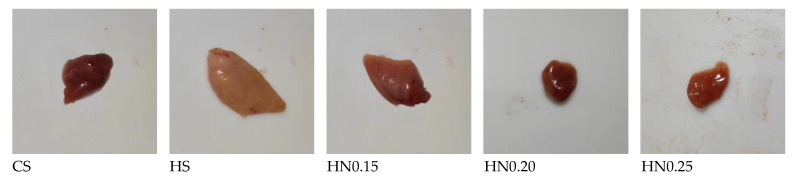
Liver morphology of largemouth bass in the five treatment groups.

The results of HE-stained liver sections are shown in Figure 15. In the HS group, extensive hepatocyte vacuolation, blurred cell boundaries, nuclear disappearance, and inflammatory cell infiltration were observed. In the CS, HN0.15, HN0.20, and HN0.25 groups, the hepatocyte structure was clearer, nuclei were visible, and obvious vacuolation was not observed. Mild inflammatory cell infiltration was observed in the HN0.15 and HN0.20 groups. Quantitative analysis showed that the vacuolated area in the HN0.15, HN0.20, and HN0.25 groups was not significantly different from that in the CS group (*p* > 0.05), but was significantly lower than that in the HS group (*p* < 0.05; Figure 16). As shown in Figure 17, the liver color in the HS group was distinctly paler than that in the other groups.

## 4. Discussion

With the rising cost of protein and fat sources, starch has become a popular ingredient in aquafeeds. However, excessive dietary starch intake may lead to various adverse effects in carnivorous fish with limited starch tolerance [39]. Largemouth bass is a typical example of poor starch utilization, and its growth performance is reduced at dietary starch levels above 15% [40]. Similarly, largemouth bass in the HS group exhibited significantly reduced growth performance compared to those in the CS group. Largemouth bass in the HN0.15, HN0.20, and HN0.25 groups showed improved growth, but could not recover to the level of the CS group. NCG improved the growth of carp (Cyprinus carpio) on arginine-deficient diets [32]. The expression of the *asl* and *cps-1* genes involved in arginine synthesis in the gut was upregulated in the HN0.15 and HN0.20 groups, accompanied by a significant increase in body protein proportion. This upregulation may be attributed to NCG promoting endogenous arginine synthesis [22]. Arginine helps insulin-like growth factor and growth hormone fulfill their roles [41]. Previous studies have suggested that arginine may participate in glucose utilization, growth regulation, and protein deposition through AMPK- and TOR-related metabolic responses [42,43,44].

### 4.1. Regulation of Glycolipid Metabolism by NCG

Largemouth bass fed the HS diet not only exhibited reduced growth performance but also showed an increased hepatosomatic index. Serum TG levels were significantly increased in the HS group, indicating that high-starch feeding disturbed circulating lipid metabolism [7,9,16,21]. However, hepatic TG content in the HS group was not significantly different from that in the CS group, and Oil Red O staining showed no obvious lipid droplet deposition among the five groups. These results indicate that the elevated serum TG did not correspond to marked hepatic lipid deposition under the present experimental conditions. Instead, hepatic glycogen content was increased in the HS group, suggesting the development of glycogen-related hepatic alterations. The increased hepatosomatic index and hepatocyte vacuolation further suggested that the primary hepatic alteration induced by the high-starch diet was glycogen accumulation rather than lipid deposition [17,18,39]. The hepatosomatic index and hepatic glycogen levels of fish in the HN0.20 and HN0.25 groups did not differ from those of the CS group, suggesting that dietary NCG alleviated high-starch-induced hepatic glycogen accumulation.

Largemouth bass in the HN0.15, HN0.20, and HN0.25 groups showed significantly elevated serum NO levels, along with upregulated expression of insulin-signaling-related genes, including *insr* and *irs*, and glycolysis-related genes, including *gk* and *pk*. This may be attributed to the ability of NCG to promote endogenous arginine synthesis [22] and insulin secretion [45]. NO has been reported to participate in the regulation of glucose and lipid metabolism in insulin-sensitive tissues [46,47,48]. These results suggest that dietary NCG may be associated with improved glucose utilization and reduced hepatic glycogen accumulation in largemouth bass fed a high-starch diet. In addition, increased serum NO levels and upregulated expression of *atgl*, *hsl*, and *leptin* were observed in the NCG-supplemented groups. Serum and hepatic TG levels were also lower in these groups than in the HS group. These results suggest that NCG improved lipid metabolic status, which may be related to enhanced transcriptional responses associated with TG catabolism and NO–leptin regulation [49,50,51].

### 4.2. Alleviation of Glycogenic Hepatopathy by NCG

Fish that consume high-starch diets often develop glycogenic hepatopathy due to excess glycogen in the liver cells, leading to liver enlargement [18]. In human glycogenic hepatopathy, there is typically no significant inflammation or fibrosis in the liver [52]. However, in fish experiencing prolonged stress from hepatic glycogen accumulation, inflammation, apoptosis, and fibrosis may occur [53]. Higher glycogen content in the fish liver has been associated with lower antioxidant capacity [54]. In the present study, largemouth bass in the HS group showed increased expression of inflammatory and apoptotic genes and elevated MDA content, along with decreased hepatic GSH-PX and SOD activities compared with the CS group. Histological analyses of hepatic hematoxylin–eosin staining, as well as serum alanine aminotransferase (ALT) and aspartate aminotransferase (AST) activities, showed obvious hepatocyte vacuolation and hepatic injury, suggesting the development of high-starch-induced glycogen-related hepatopathy in largemouth bass. In the present study, the HS group showed a significantly higher mortality rate, particularly toward the end of the experimental period. The increased mortality in the HS group may be related to the prolonged metabolic stress and hepatic injury associated with excessive dietary starch intake, although the direct cause of mortality requires further investigation.

Previous studies have suggested that the mTOR signaling pathway is involved in various forms of liver injury, including fatty liver injury and thioacetamide-induced liver injury [55,56]. In the present study, *mtor* gene expression was increased in the HS group, suggesting that mTOR-related transcriptional responses may be associated with high-starch-induced hepatic injury. Studies in the mouse liver have reported that reduced mtor expression is associated with lower expression of apoptotic factors, reduced NF-κB-related inflammatory responses, and enhanced autophagy-related processes [57,58,59]. The expression of mtor was reduced by the addition of 0.15%, 0.20%, and 0.25% NCG to the high-starch diet. A similar down-regulation of mtor-related responses by NCG has also been reported in Japanese sea bass (*Lateolabrax japonicus*) [60]. Largemouth bass in HN0.15, HN0.20 and HN0.25 groups showed significantly lower expression of hepatic inflammatory and apoptotic genes, increased antioxidant enzyme activities to varying degrees, and lower serum ALT and AST, which were not significantly different from the CS group. In rats, increased NO levels have been reported to modulate inflammatory cytokine secretion and downregulate inflammatory gene expression, possibly involving guanylate cyclase-related signaling [61,62]. These findings suggest that the protective effects of dietary NCG may be associated with reduced *mtor* expression and improved NO-related metabolic regulation.

Arginine has been reported to influence genes related to GSH metabolism and antioxidant defense, possibly involving nrf2-related regulation in rats [63]. In several fish studies, arginine supplementation has been associated with increased SOD and GSH-PX activities, together with upregulated *nrf2* expression and downregulated *keap1* expression [64,65,66]. Similarly, in the present study, dietary NCG was associated with increased antioxidant enzyme activities, upregulated *nrf2* expression, and downregulated *keap1* expression, suggesting a possible involvement of *Nrf2/Keap1*-related transcriptional regulation.

The addition of 0.20%, and 0.25% NCG to high-starch diets alleviated hepatic glycogen accumulation and liver injury in largemouth bass. These beneficial effects were accompanied by increased serum NO levels, reduced mtor expression, improved hepatic antioxidant capacity, upregulated *nrf2* expression, downregulated *keap1* expression, and lower expression of inflammatory and apoptotic genes.

## 5. Conclusions

In conclusion, dietary NCG supplementation partially mitigated high-starch-induced growth suppression, hepatic glycogen accumulation, oxidative stress, and liver injury in largemouth bass. These beneficial effects were accompanied by increased serum NO levels, improved glucose and lipid metabolic status, enhanced antioxidant capacity, and reduced transcription of inflammation- and apoptosis-related genes. At the transcriptional level, NCG supplementation modulated the expression of genes involved in insulin signaling, glycolysis, lipid catabolism, antioxidant regulation, and mTOR/NF-κB-related inflammatory responses. Collectively, these findings indicate that dietary NCG contributes to maintaining hepatic metabolic homeostasis and redox balance under high-starch feeding conditions and may serve as a promising nutritional strategy for improving the tolerance of largemouth bass to high-starch diets.

## Figures and Tables

**Table 1:** Formulation and analyzed proximate composition of the experimental diets’ % dry matter.

Ingredients (%)	CS	HS	HN0.15	HN0.20	HN0.25
fish meal	51.00	51.00	51.00	51.00	51.00
chicken meal	8.00	8.00	8.00	8.00	8.00
plasma protein flour	4.00	4.00	4.00	4.00	4.00
vital wheat gluten	3.42	3.42	3.42	3.42	3.42
cassava starch	9.00	9.00	9.00	9.00	9.00
wheat flour	4.00	4.00	4.00	4.00	4.00
seaweed powder	4.00	4.00	4.00	4.00	4.00
fish oil	4.00	4.00	4.00	4.00	4.00
soybean oil	1.00	1.00	1.00	1.00	1.00
lysophosphalipid	0.10	0.10	0.10	0.10	0.10
choline chloride	0.30	0.30	0.30	0.30	0.30
vitamin mixturea	1.00	1.00	1.00	1.00	1.00
vitamin C	0.03	0.03	0.03	0.03	0.03
mineral mixtureb	3.00	3.00	3.00	3.00	3.00
l-methionine	0.40	0.40	0.40	0.40	0.40
corn starch	0.00	6.50	6.50	6.50	6.50
NCG	0.00	0.00	0.15	0.20	0.25
microcrystalline cellulose	5.75	0.25	0.10	0.05	0.00
total	100.00	100.00	100.00	100.00	100.00
proximate composition					
moisture	9.74	10.11	9.96	10.41	10.07
crude protein	47.85	48.10	47.96	47.96	48.04
crude lipid	11.55	11.53	11.55	11.58	11.60
starch	11.50	18.00	18.00	18.00	18.00
ash	13.24	13.54	13.41	13.43	13.48

Vitamin premix (mg/kg of diet): vitamin B1, 800; vitamin B2, 1600; vitamin B6, 1200; nicotinic acid, 3800; D-Ca pantothenate, 2500; myo-inositol, 4000; d-biotin, 40; folic acid, 320; vitamin A, 400,000 IU; vitamin E, 16,000; vitamin K3, 600; vitamin D3, 80,000 IU; vitamin B12, 4; vitamin C, 35,000. Mineral premix (mg/kg of diet): magnesium, 3000; iron, 1800; manganese, 800; iodine, 250; copper, 350; zinc, 6500; selenium, 15; cobalt, 60. Ingredient composition values are calculated percentages. Proximate composition values represent the analyzed composition of each homogeneous diet batch.

**Table 2:** Methods of analyzing the composition of the feed and fish.

	Method	Standard Number
Moisture (%)	Constant weight method	(GB/T 6435-2006) [34]
Crude lipid (%)	Soxhlet extraction method	(GB/T 6433-2006) [35]
Crude protein (%)	Micro Kjeldahl method	(GB/T 6432-2018) [36]
Ash (%)	Constant weight method	(GB/T 6438-2007) [37]

**Table 3:** Primers used for qRT-PCR analysis.

Gene	Primer Sequence (5′–3′)	Accession Number	Amplicon Size (bp)	Amplification Efficiency (%)
*β-actin*	F: AAAGGGAAATCGTGCGTGAC	XM_038695351.1	136	98
	R: AAGGAAGGCTGGAAGAGGG			
*ef1α*	F: TGCTGCTGGTGTTGGTGAGTT	XM_038695471.1	147	95
	R: TTCTGGCTGTAAGGGGGCTC			
*caspase-3*	F: GCTTCATTCGTCTGTGTTC	NW_024044237.1	98	102
	R: CGAAAAAGTGATGTGAGGTA			
*caspase-8*	F: TGTTCACCCACCTTGGCTTT	NW_024040485.1	90	102
	R: CCCTTCCGCTGAGGTCTTTT			
*caspase-9*	F: ATCCACGAGGGAGACAAAGAG	NW_024040707.1	97	108
	R: GCAACCGAGCACAAATAAGAG			
*bad*	F: CACATTTCGGATGCCACTAT	NW_024041150.1	116	101
	R: TTCTGCTCTTCTGCGATTGA			
*p53*	F: AGATTGAATGGTGGTGGG	NW_024041039.1	144	105
	R: GTTCTGGCGGACTGGA			
*il-15*	F: GTATGCTGCTTCTGTGCCTGG	NW_024042261.1	82	101
	R: AGCGTCAGATTTCTCAATGGTGT			
*tnf-α*	F: CTTCGTCTACAGCCAGGCATCG	NW_024040817.1	161	108
	R: TTTGGCACACCGACCTCACC			
*Il-8*	F: GCTCAAAGAGAGCGAGGATG	NW_024040928.1	118	105
	R: TCCTCTACCATTCGCAATCC			
*tgf-β*	F: CGTTGAACAGACTGGGAGAGATG	NW_024044459.1	112	110
	R: AGTGGGATGGCTTCATTATCTTGT			
*fas*	F: TTACACTGCCACAGCAACCA	XM_038693765.1	92	95
	R: TGCCCCTCCTACTACACCTC			
*acc*	F: TAGTCCAGTGCCCATCCTCA	NW_024044681.1	96	98
	R: CCAGAAAAGCCCCTCCAGTT			
*atgl*	F: CCATGATGCTCCCCTACACT	XM_038705351.1	176	101
	R: GGCAGATACACTTCGGGAAA			
*hsl*	F: ATCAGAGCTGGAGCACCCTA	XM_038725628.1	122	110
	R: GCAGAGGAGAGCAGAAAGGA			
*gp*	F: ACAGAGTGGTGGACGAGACC	NW_024040040.1	115	95
	R: TCGTTCACCAGCTTCATCAG			
*pk*	F: GCTGAAAAGGAAACACCAAAG	NW_024040152.1	152	108
	R: ACAGCCGTAGACCCAATAGA			
*pepck*	F: TCCATCCATCGTCAACCGCTTA	NW_024043372.1	119	90
	R: ACACCGCCATCGCTAGTCTCT			
*g6pase*	F: GGGAGTCCAGGTGTGTGTCT	NW_024041262.1	182	95
	R: CAGCGAAGGAGGTCAAGAAG			
*insr*	F: CCTCCGCACAGCAGTCAGATTC	XM_038715865.1	198	101
	R: AGCAGCCACAGTCATAACCACAAT			
*irs*	F: AGGCGGAGGATTCTGTGG	XM_038709651.1	150	91
	R: TGAGGTTGCGTCGTGTGG			
*nrf2*	F: TCACCAAAGACAAGCGTAA	XM_038720536.1	111	105
	R: CAGGCAGATTGATAATCATAGA			
*ampk*	F: TGTCACATCTCATCACTCTG	Cluster-21914.16926	147	100
	R: TGTAGAAGACGCCTCCTC			
*NF-κB*	F: TGATGATAACTGGCTTCGG	NW_024044237.1	86	104
	R: TCAAACCTGGACCCTACCT			
*leptin*	F: GGACAAAGACTTCCAGGTCCC	NW_024044570.1	117	104
	R: ACCCTCCAAGACGGTCACTA			
*mtor*	F: CCATCCTCAACCTACTTCC	XM_038733437.1	105	92
	R: CTCTCCTTCTCCTTCTTCAG			
*keap1*	F: CCTGTTGCATCAGTTGTGCC	NW_024041150.1	126	93
	R: GAGCCCTCGTGGGAAAGAAA			
*asl*	F: CCATCTCAACCCTGACGGAC	XM_038709368.1	131	104
	R: CACAACGAAGCCCAGAACAA			
*cps-1*	F: CGCTCCGTCTTCTCCAACATACTG	XM_038720291.1	139	106
	R: TGCCGCTCCACTACACTCACA			
*p5cs*	F: GCCTAGAGATTCACACCACGACTAT	XM_038738134.1	182	97
	R: CCACCGATTAAGCATTACACTTCCAT			

**Table 4:** Effects of NCG added in a high-starch diet on the growth performance of largemouth bass.

	CS	HS	HN0.15	HN0.20	HN0.25
IBW	5.96 ± 0.01	5.96 ± 0.01	5.96 ± 0.01	5.96 ± 0.01	5.96 ± 0.01
FBW	37.19 ± 0.35 a	30.01 ± 0.17 d	33.38 ± 0.18 b	33.97 ± 0.33 b	31.60 ± 0.39 c
WGR	516.16 ± 1.67 a	313.32 ± 6.17 c	412.87 ± 2.56 b	443.96 ± 10.79 b	405.83 ± 14.56 b
SGR	3.38 ± 0.02 a	2.99 ± 0.01 d	3.19 ± 0.031 b	3.22 ± 0.02 b	3.09 ± 0.02 c
SR	96.67 ± 0.00 a	83.34 ± 0.00 b	93.34 ± 0.00 a	96.67 ± 0.00 a	96.67 ± 0.00 a
FI	0.84 ± 0.01 ab	0.87 ± 0.01 a	0.79 ± 0.03 b	0.78 ± 0.01 b	0.81 ± 0.01 ab

Note: Initial body weight (IBW, g); final body weight (FBW, g) = final total weight/final number of fish; weight gain rate (WGR, %) = 100 × (FBW − IBW)/IBW; specific growth rate (SGR, %/day) = 100 × (Ln FBW − Ln IBW)/number of feeding days; survival rate (SR, %) = 100 × (final number of fish)/(initial number of fish); feed intake (FI) = 100 × feed consumption (g)/[(final number of fish)/(initial number of fish)]/2. Values are mean ± SEM (n = 3). Data with different letters were significantly different between all treatment groups (*p* < 0.05). One-way ANOVA results: FBW, F(4, 10) = 82.53; WGR, F(4, 10) = 71.28; SGR, F(4, 10) = 80.98; SR, F(4, 10) = 12.77; FI, F(4, 10) = 5.23.

**Table 5:** Effects of NCG added in a high-starch diet on the fish composition of largemouth bass.

	CS	HS	HN0.15	HN0.20	HN0.25
Moisture (%)	64.81 ± 0.33	65.07 ± 0.20	65.33 ± 0.84	66.18 ± 0.28	65.38 ± 0.71
Crude protein (%)	23.87 ± 0.04 c	24.53 ± 0.26 bc	27.29 ± 0.92 a	27.72 ± 0.81 a	27.06 ± 0.02 ab
Crude lipid (%)	9.32 ± 0.23	9.65 ± 0.05	9.65 ± 0.18	9.52 ± 0.07	9.49 ± 0.13
Ash (%)	4.59 ± 0.01	4.62 ± 0.03	4.52 ± 0.04	4.43 ± 0.21	4.47 ± 0.09

Values are mean ± SEM (n = 3). Data with different letters were significantly different between all treatment groups (*p* < 0.05). One-way ANOVA results: Moisture, F(4, 10) = 94.12; crude protein, F(4, 10) = 9.86; crude lipid, F(4, 10) = 0.89; ash, F(4, 10) = 0.61.

## Data Availability

The original data presented in the study are openly available in Zenodo at https://doi.org/10.5281/zenodo.19878268.

## References

[1] NRC (2011). Carbohydrates and Fibre. Nutrient Requirements of Fish and Shrimp.

[2] Guo J., Kuang W., Zhong Y., Zhou Y., Chen Y., Lin S. (2020). Effects of supplemental dietary bile acids on growth, liver function and immunity of juvenile largemouth bass (*Micropterus salmoides*) fed high-starch diet. Fish Shellfish Immunol..

[3] Azaza M.S., Khiari N., Dhraief M.N., Aloui N., Kraϊem M.M., Elfeki A. (2015). Growth performance, oxidative stress indices and hepatic carbohydrate metabolic enzymes activities of juvenile Nile tilapia, *Oreochromis niloticus* L., in response to dietary starch to protein ratios. Aquac. Res..

[4] Honorato C.A., Almeida L.C., Da Silva Nunes C., Carneiro D.J., Moraes G. (2010). Effects of processing on physical characteristics of diets with distinct levels of carbohydrates and lipids: The outcomes on the growth of pacu (*Piaractus mesopotamicus*). Aquacult. Nutr..

[5] Li X., Wang J., Han T., Hu S., Jiang Y. (2015). Effects of dietary carbohydrate level on growth and body composition of juvenile giant croaker *Nibea japonica*. Aquac. Res..

[6] Zhou C., Ge X., Niu J., Lin H., Huang Z., Tan X. (2015). Effect of dietary carbohydrate levels on growth performance, body composition, intestinal and hepatic enzyme activities, and growth hormone gene expression of juvenile golden pompano, *Trachinotus ovatus*. Aquaculture.

[7] Kamalam B.S., Medale F., Panserat S. (2017). Utilisation of dietary carbohydrates in farmed fishes: New insights on influencing factors, biological limitations and future strategies. Aquaculture.

[8] Tian L.X., Liu Y.J., Yang H.J., Liang G.Y., Niu J. (2012). Effects of different dietary wheat starch levels on growth, feed efficiency and digestibility in grass carp (*Ctenopharyngodon idella*). Aquacult. Int..

[9] Lin S., Shi C., Mu M., Chen Y., Luo L. (2018). Effect of high dietary starch levels on growth, hepatic glucose metabolism, oxidative status and immune response of juvenile largemouth bass, *Micropterus salmoides*. Fish Shellfish Immunol..

[10] Li X.F., Wang Y., Liu W.B., Jiang G.Z., Zhu J. (2013). Effects of dietary carbohydrate/lipid ratios on growth performance, body composition and glucose metabolism of fingerling blunt snout bream *Megalobrama amblycephala*. Aquacult. Nutr..

[11] Russell P.M., Davies S.J., Gouveia A., Tekinay A.A. (2001). Influence of dietary starch source on liver morphology in juvenile cultured European sea bass (*Dicentrarchus labrax* L.). Aquac. Res..

[12] Liu Y., Liu N., Wang A., Chen N., Li S. (2022). Resveratrol inclusion alleviated high-dietary-carbohydrate-induced glycogen deposition and immune response of largemouth bass, *Micropterus salmoides*. Brit. J. Nutr..

[13] Wu C., Ye J., Gao J., Chen L., Lu Z. (2016). The effects of dietary carbohydrate on the growth, antioxidant capacities, innate immune responses and pathogen resistance of juvenile Black carp *Mylopharyngodon piceus*. Fish Shellfish Immunol..

[14] Gatesoupe F., Huelvan C., Le Bayon N., Sévère A., Aasen I.M., Degnes K.F., Mazurais D., Panserat S., Zambonino-Infante J.L., Kaushik S.J. (2014). The effects of dietary carbohydrate sources and forms on metabolic response and intestinal microbiota in sea bass juveniles, *Dicentrarchus labrax*. Aquaculture.

[15] Rural Ministry of Agriculture Fisheries and Fisheries Administration, China Fisheries Society (2021). 2021 China Fisheries Statistics Yearbook.

[16] Liang X., Chen P., Wu X., Xing S., Morais S., He M., Gu X., Xue M. (2022). Effects of High Starch and Supplementation of an Olive Extract on the Growth Performance, Hepatic Antioxidant Capacity and Lipid Metabolism of Largemouth Bass (*Micropterus salmoides*). Antioxidants.

[17] Zhang Y., Xie S., Wei H., Zheng L., Liu Z., Fang H., Xie J., Liao S., Tian L., Liu Y. (2020). High dietary starch impaired growth performance, liver histology and hepatic glucose metabolism of juvenile largemouth bass, *Micropterus salmoides*. Aquacult. Nutr..

[18] Li X., Han T., Zheng S., Wu G. (2022). Hepatic Glucose Metabolism and Its Disorders in Fish. Adv. Exp. Med. Biol..

[19] Li X., Zheng S., Ma X., Cheng K., Wu G. (2020). Effects of dietary starch and lipid levels on the protein retention and growth of largemouth bass (*Micropterus salmoides*). Amino Acids.

[20] Feng Z., Zhong Y., He G., Sun H., Chen Y., Zhou W., Lin S. (2022). Yeast culture improved the growth performance, liver function, intestinal barrier and microbiota of juvenile largemouth bass (*Micropterus salmoides*) fed high-starch diet. Fish Shellfish Immunol..

[21] Wang T., Xu R., Qiao F., Du Z., Zhang M. (2022). Effects of mannan oligosaccharides (MOS) on glucose and lipid metabolism of largemouth bass (*Micropterus salmoides*) fed with high carbohydrate diet. Anim. Feed Sci. Tech..

[22] Wu G., Knabe Darrell A., Ki S.W. (2004). Arginine Nutrition in Neonatal Pigs. J. Nutr..

[23] Schwahn B.C., Pieterse L., Bisset W.M., Galloway P.G., Robinson P.H. (2010). Biochemical efficacy of N-carbamylglutamate in neonatal severe hyperammonaemia due to propionic acidaemia. Eur. J. Pediatr..

[24] Cao W., Xiao L., Liu G., Fang T., Wu X., Jia G., Zhao H., Chen X., Wu C., Cai J. (2016). Dietary arginine and N-carbamylglutamate supplementation enhances the antioxidant statuses of the liver and plasma against oxidative stress in rats. Food Funct..

[25] Wu X., Ruan Z., Gao Y., Yin Y., Zhou X., Wang L., Geng M., Hou Y., Wu G. (2010). Dietary supplementation with l-arginine or N-carbamylglutamate enhances intestinal growth and heat shock protein-70 expression in weanling pigs fed a corn- and soybean meal-based diet. Amino Acids.

[26] Frank J.W., Escobar J., Nguyen H.V., Jobgen S.C., Jobgen W.S., Davis T.A., Wu G. (2007). Oral N-Carbamylglutamate Supplementation Increases Protein Synthesis in Skeletal Muscle of Piglets1. J. Nutr..

[27] Wang L., Wu J., Wang C., Li J., Zhao Z., Luo L., Du X., Xu Q. (2017). Dietary arginine requirement of juvenile hybrid sturgeon (*Acipenser schrenckii* ♀ × *Acipenser baerii* ♂). Aquac. Res..

[28] Du Z.Y., Liu Y.J., Tian L.X., Wang J.T., Wang Y., Liang G.Y. (2005). Effect of dietary lipid level on growth, feed utilization and body composition by juvenile grass carp (*Ctenopharyngodon idella*). Aquacult. Nutr..

[29] Cheng W., Zhang L., Xu G., Wu Q., Xiong D., Guo Y., Tan W. (2015). Effects of arginine on the regulation of the growth, the blood amino acid position and the fat deposition in nile tilapia (*Oreochromis Niloticus*). Acta Hydrobiol. Sin..

[30] Huang H., Zhang X., Liang X., Wu X., Gu X., Han J., Xue M. (2021). N-carbamoylglutamate improves lipid metabolism, inflammation, and apoptosis responses in visceral adipocytes of Japanese seabass (*Lateolabrax japonicus*), in vivo and in vitro. Anim. Nutr..

[31] Leclercq-Meyer V., Marchand J., Malaisse W.J. (1990). Stimulus-secretion coupling of arginineinduced insulin release: Resistance of arginine- and ornithine-stimulated glucagon and insulin release to d,l-α-difluoromethylornithine. Biochem. Pharmacol..

[32] Wang L., Li J., Wang C., Zhao Z., Luo L., Du X., Xu Q. (2019). Effect of N-carbamoylglutamate supplementation on the growth performance, antioxidant status and immune response of mirror carp (*Cyprinus carpio*) fed an arginine-deficient diet. Fish Shellfish Immunol..

[33] Cheng T., Chen J., Tan B., Chi S. (2025). Effects of α-lipoic acid (LA) supplementation in high-fat diet on the growth, glycolipid metabolism and liver health of largemouth bass (*Micropterus salmoides*). Fish Shellfish Immunol..

[34] (2006). Determination of Moisture and Other Volatile Mater Content in Feeds.

[35] (2006). Determination of Crude Fat in Feeds.

[36] (2018). Determination of Crude Protein in Feeds—Kjeldahl Method.

[37] (2007). Animal Feeding Stuffs—Determination of Crude Ash.

[38] Livak K.J., Schmittgen T.D. (2001). Analysis of relative gene expression data using real-time quantitative PCR and the 2^−ΔΔCt^ method. Methods.

[39] Zhong L., Liu H., Zhang H., Zhang W., Li M., Huang Y., Yao J., Huang X., Geng Y., Chen D. (2022). High Starch in Diet Leads to Disruption of Hepatic Glycogen Metabolism and Liver Fibrosis in Largemouth Bass (*Micropterus salmoides*), Which is Mediated by the PI3K/Akt Signaling Pathway. Front. Physiol..

[40] Ma H., Mou M., Pu D., Lin S., Chen Y., Luo L. (2019). Effect of dietary starch level on growth, metabolism enzyme and oxidative status of juvenile largemouth bass, *Micropterus salmoides*. Aquaculture.

[41] Lall S.P., Kaushik S.J., Le Bail P.Y., Keith R., Anderson J.S., Plisetskaya E. (1994). Quantitative arginine requirement of Atlantic salmon (*Salmo salar*) reared in sea water. Aquaculture.

[42] Berge G.E., Lied E., Sveier H. (1997). Nutrition of Atlantic Salmon (*Salmo salar*): The Requirement and Metabolism of Arginine. Comp. Biochem. Physiol. Part A Physiol..

[43] Liang H., Ren M., Habte-Tsion H., Ge X., Xie J., Mi H., Xi B., Miao L., Liu B., Zhou Q. (2016). Dietary arginine affects growth performance, plasma amino acid contents and gene expressions of the TOR signaling pathway in juvenile blunt snout bream, *Megalobrama amblycephala*. Aquaculture.

[44] Wang B., Liu Y., Feng L., Jiang W., Kuang S., Jiang J., Li S., Tang L., Zhou X. (2015). Effects of dietary arginine supplementation on growth performance, flesh quality, muscle antioxidant capacity and antioxidant-related signalling molecule expression in young grass carp (*Ctenopharyngodon idella*). Food Chem..

[45] Andoh T. (2007). Amino acids are more important insulinotropins than glucose in a teleost fish, barfin flounder (*Verasper moseri*). Gen. Comp. Endocr..

[46] Mommsen T.P., Moon T.W., Plisetskaya E.M. (2001). Effects of arginine on pancreatic hormones and hepatic metabolism in rainbow trout. Physiol. Biochem. Zool..

[47] Li P., Mai K., Trushenski J., Wu G. (2009). New developments in fish amino acid nutrition: Towards functional and environmentally oriented aquafeeds. Amino Acids.

[48] Jobgen W.S., Fried S.K., Fu W.J., Meininger C.J., Wu G. (2006). Regulatory role for the arginine–nitric oxide pathway in metabolism of energy substrates. J. Nutr. Biochem..

[49] Fu W.J., Haynes T.E., Kohli R., Hu J., Shi W., Spencer T.E., Carroll R.J., Meininger C.J., Wu G. (2005). Dietary L-arginine supplementation reduces fat mass in Zucker diabetic fatty rats. J. Nutr..

[50] Frühbeck G., Gómez-Ambrosi J. (2001). Modulation of the leptin-induced white adipose tissue lipolysis by nitric oxide. Cell. Signal..

[51] Yu W.H., Kimura M., Walczewska A., Karanth S., Mccann S.M. (1997). Role of leptin in hypothalamic-pituitary function. Proc. Natl. Acad. Sci. USA.

[52] Torbenson M., Chen Y., Brunt E., Cummings O.W., Gottfried M., Jakate S., Liu Y., Yeh M.M., Ferrell L. (2006). Glycogenic Hepatopathy: An Under-recognized Hepatic Complication of Diabetes Mellitus. Am. J. Surg. Pathol..

[53] Li X., Shixuan Z., Jia S., Song F., Zhou C., Wu G. (2020). Oxidation of energy substrates in tissues of largemouth bass (*Micropterus salmoides*). Amino Acids.

[54] Lygren B., Hemre G. (2001). Influence of dietary carbohydrate on antioxidant enzyme activities in liver of Atlantic salmon (*Salmo salar L.*). Aquacult. Int..

[55] Abdelrahman R.S., El-Tanbouly G.S. (2022). Protocatechuic acid protects against thioacetamide-induced chronic liver injury and encephalopathy in mice via modulating mTOR, p53 and the IL-6/IL-17/IL-23 immunoinflammatory pathway. Toxicol. Appl. Pharm..

[56] Feng J., Qiu S., Zhou S., Tan Y., Bai Y., Cao H., Guo J., Su Z. (2022). mTOR: A Potential New Target in Nonalcoholic Fatty Liver Disease. Int. J. Mol. Sci..

[57] Nasiri-Ansari N., Nikolopoulou C., Papoutsi K., Kyrou I., Mantzoros C.S., Kyriakopoulos G., Chatzigeorgiou A., Kalotychou V., Randeva M.S., Chatha K. (2021). Empagliflozin Attenuates Non-Alcoholic Fatty Liver Disease (NAFLD) in High Fat Diet Fed ApoE(-/-) Mice by Activating Autophagy and Reducing ER Stress and Apoptosis. Int. J. Mol. Sci..

[58] Luo J., Li Y., Zhai Y., Liu Y., Zeng J., Wang D., Li L., Zhu Z., Chang B., Deng F. (2022). D-Mannose ameliorates DNCB-induced atopic dermatitis in mice and TNF-α-induced inflammation in human keratinocytes via mTOR/NF-κB pathway. Int. Immunopharmacol..

[59] Sun C., Zhang J., Hou J., Hui M., Qi H., Lei T., Zhang X., Zhao L., Du H. (2023). Induction of autophagy via the PI3K/Akt/mTOR signaling pathway by Pueraria flavonoids improves non-alcoholic fatty liver disease in obese mice. Biomed. Pharmacother..

[60] Huang H.Y., Chen P., Liang X.F., Wu X.F., Wang C.P., Gu X., Xue M. (2019). Dietary N-Carbamylglutamate (NCG) alleviates liver metabolic disease and hepatocyte apoptosis by suppressing ERK1/2-mTOR-S6K1 signal pathway via promoting endogenous arginine synthesis in Japanese seabass (*Lateolabrax japonicus*). Fish Shellfish Immunol..

[61] Boczkowski J., Philipp I., Tedgui A., Bernard C., Merval R., Desmonts J., Aubier M. (1995). Effects of inhibition of nitric oxide synthesis on TNFα serum levels in e. coli endotoxemic rats. Life Sci..

[62] Mo W., Wu X., Jia G., Zhao H., Chen X., Tang J., Wu C., Cai J., Tian G., Wang J. (2018). Roles of dietary supplementation with arginine or N-carbamylglutamate in modulating the inflammation, antioxidant property, and mRNA expression of antioxidant-relative signaling molecules in the spleen of rats under oxidative stress. Anim. Nutr..

[63] Liang M., Wang Z., Li H., Cai L., Pan J., He H., Wu Q., Tang Y., Ma J., Yang L. (2018). l-Arginine induces antioxidant response to prevent oxidative stress via stimulation of glutathione synthesis and activation of Nrf2 pathway. Food Chem. Toxicol..

[64] Yu Y., Huang D., Zhang L., Chen X., Wang Y., Zhang L., Ren M., Liang H. (2023). Dietary arginine levels affect growth performance, intestinal antioxidant capacity and immune responses in largemouth bass (*Micropterus salmoides*). Aquac. Rep..

[65] Li P., Hou D., Zhao H., Peng K., Chen B., Guo H., Cao J. (2022). Effects of dietary arginine levels on intestinal morphology, digestive enzyme activity, antioxidant capacity and intestinal flora of hybrid snakehead(*Channa maculata* ♀ × *Channa argus* ♂). Aquac. Rep..

[66] Liang H., Mokrani A., Ji K., Ge X., Ren M., Pan L., Sun A. (2018). Effects of dietary arginine on intestinal antioxidant status and immunity involved in Nrf2 and NF-κB signaling pathway in juvenile blunt snout bream, *Megalobrama amblycephala*. Fish. Shellfish Immunol..

